# Exploring Mental Health Awareness: A Study on Knowledge and Perceptions of Mental Health Disorders among Residents of Matsafeni Village, Mbombela, Mpumalanga Province

**DOI:** 10.3390/healthcare12010085

**Published:** 2023-12-29

**Authors:** Eseldah Nkhensani Mboweni, Mabitsela Hezekiel Mphasha, Linda Skaal

**Affiliations:** 1 Department of Public Health, University of Limpopo, Mankweng 0727, South Africa; 200405323@keyaka.ul.ac.za; 2 Department of Human Nutrition and Dietetics, University of Limpopo, Mankweng 0727, South Africa; 3 Department of Public Health, Sefako Makgatho University, Ga-Rankuwa 0204, South Africa; skaal651@gmail.com

**Keywords:** perception, knowledge, community, mental health, mental disorder

## Abstract

The global rise in mental health disorders has significant social, economic, and physical impacts. Despite advancements in support, cultural beliefs attributing mental illnesses to spiritual causes persist, fostering discrimination and stigmatization. The study aims to explore the understanding and perceptions of mental health in Matsafeni Village, acknowledging the complexity of mental health issues. A qualitative method and a descriptive exploratory design were employed, enabling the researcher to describe, examine, and explore the knowledge and perceptions regarding mental health. Data collection was conducted through unstructured, open-ended interviews, with 15 participants selected through convenience sampling. The data were analyzed through thematic analysis. Measures of rigor were ensured through credibility, transferability, confirmability, and dependability. Participants demonstrated knowledge of mental health disorders, recognizing disruptions in thought patterns and diverse symptoms. They highlighted key signs and behaviors, emphasizing the need for spotting indicators such as untidiness. Perceptions of the causes of mental illness varied, including witchcraft and genetics. Participants unanimously advocated for seeking help from traditional healers, medical facilities, and therapies. Community members shared their views of mental health, covering their understanding, recognition of signs, personal interactions, and observations of behaviors in individuals with mental health conditions. Reported symptoms align with existing research, emphasizing the complexity of managing safety concerns in severe mental illnesses. The study highlights the need for community education to reduce stigma, considering cultural factors in mental health perceptions. Recommendations include early interventions, enhanced mental health services, and collaboration between western and traditional approaches for a holistic and culturally sensitive approach to mental health.

## 1. Background

Mental health disorders pose a critical global public health concern, affecting individuals and communities worldwide [1]. The prevalence of anxiety and depressive disorders has risen, impacting one in eight people globally in 2019 [2]. Alarming statistics reveal that only one in three individuals facing depression receives adequate care, emphasizing the urgent need for enhanced global mental health support [3]. In Africa, mental health problems contribute to 19% of disabilities, affecting one in four Africans, leading to substantial productivity loss. The prevalence of depression in Africa reached 116.29 million in 2019, with South Africa bearing a considerable burden. In low- and middle-income countries like South Africa, factors such as conflict, trauma, hunger, poverty, limited access to health care, and social inequality contribute to the high prevalence of mental health disorders [4]. Despite the significant impact of mental disorders, these countries allocate less than 3% of their health budgets to mental health care, emphasizing the critical need for increased investment and resources in mental health initiatives [5].

South Africa specifically faces a significant mental health challenge, with over 12 million people grappling with mental health disorders, and a concerning 75% of them remain untreated, revealing a substantial treatment gap [6]. The 12-month prevalence estimate for common mental disorders in South Africa is 16.5%, affecting nearly one-third of the population during their lifetime. Anxiety disorders top the list as the most prevalent class of mental disorders in South Africa, affecting 15.8% of the population, followed by substance use disorders at 13.3% and mood disorders at 9.8% [4]. Shockingly, 25.7% of South Africans are likely to be depressed, with more than a quarter reporting moderate to severe symptoms of depression [7,8]. Although there is no clear indication of the prevalence of mental health disorders in the Mpumalanga province, where Matsafeni village is located, the province stands out with one of the highest rates of mental health disorders within South Africa [9].

Understanding mental health involves recognizing diverse psychological and psychiatric conditions that impact an individual’s well-being [10]. Different communities interpret mental health disorders in varied ways, influenced by their knowledge, beliefs, and perspectives [11]. Informed individuals recognize the unique manifestations of mental health disorders, allowing them to identify potential concerns in themselves or others [12]. Strong mental health literacy empowers individuals to comprehend the prevalence and impact of mental health disorders, promoting awareness and addressing the issue within society [13]. Well-informed individuals are knowledgeable about evidence-based treatments, including psychotherapy, medication, and lifestyle adjustments [14]. They are also familiar with self-help techniques, stress management, and coping mechanisms for maintaining optimal mental health. Despite progress in mental healthcare in South Africa, there remains a significant lack of awareness among individuals with mental health conditions regarding available treatment options and how to access necessary care [15]. This study aims to assess the knowledge of mental health disorders within a targeted population, intending to implement mental health literacy interventions. These interventions seek to equip individuals with the knowledge and skills needed to effectively address mental health concerns and contribute to a healthier society. Mental health literacy involves recognizing mental health disorders as medical conditions and reducing the stigma and discrimination associated with mental illness. The impact of mental health education and awareness programs extends beyond individuals to benefit society as a whole, including governments, healthcare systems, schools, workplaces, and communities. Promoting mental health awareness and education can lead to improved mental health outcomes and foster more informed and supportive attitudes within the community.

Perceptions of mental health disorders shape societal understanding and responses, with stigma being a deeply ingrained challenge [16]. Negative labels lead to discrimination and hinder help-seeking. Cultural and religious beliefs influence perceptions, creating taboos and stereotypes. Communities’ cultural heritage contributes significantly to mental health assets. Traditional practices and cultural norms actively foster the well-being of community members, providing a sense of identity, belonging, and continuity [17]. Engaging in cultural activities, including rituals, storytelling, and communal celebrations, creates a supportive environment that enhances emotional resilience and connection. Cultural engagement goes beyond tradition preservation, significantly impacting individuals’ mental well-being by reinforcing a sense of community and cultural pride. Social support is a complex and interconnected system that encompasses familial, communal, spiritual, educational, and economic networks [18]. This multifaceted structure becomes particularly evident in times of adversity, fostering shared bonds and collaborative efforts. Narrative resilience, rooted in stories of overcoming challenges, shapes the mental well-being of community members, creating a collective identity of resilience and unity [19]. These narratives, intertwined with various dimensions, serve as a source of strength, turning crises into opportunities for growth and building a lasting legacy of resilience. These narratives, intertwined with various dimensions, serve as a source of strength, turning crises into opportunities for growth and building a lasting legacy of resilience. The digital age has brought transformative changes to social support, with virtual communities and digital platforms bridging geographical gaps. Technology plays a crucial role in facilitating connections among community members, allowing them to share support regardless of physical distances [20]. Online forums, social media, and virtual gatherings not only maintain connections but also provide access to diverse perspectives and resources. This evolution in social interaction fosters inclusivity and strengthens mental well-being in the rapidly digitizing world.

Media inaccuracies perpetuate fear, hindering open discussions [21]. Positive attitudes toward mental health are crucial, fostering support and understanding. Societal awareness and education can transform perceptions, enhancing access to services and reducing discrimination [22]. Understanding mental health complexity is vital to combating misconceptions. This study in Matsafeni Village aims to explore beliefs and knowledge, addressing the community’s mental health needs. 

The investigation into mental health awareness among Matsafeni village residents in Mbombela, Mpumalanga province, is expected to uncover diverse levels of knowledge and perspectives concerning mental health disorders. The hypothesis suggests that elements such as cultural influences, educational background, social support, resilience, and accessibility to mental health resources will play a substantial role in shaping residents’ comprehension and attitudes toward mental health. Furthermore, the study foresees that its results will emphasize the necessity for specific mental health education initiatives aimed at bridging awareness gaps and fostering a more enlightened and supportive community atmosphere.

## 2. Methodological Research

### 2.1. Research Method and Design

This study used a qualitative research method with an exploratory and descriptive approach to investigate the knowledge and perceptions of mental health disorders among community members in Matsafeni Village. Individual interviews were conducted with fifteen participants, providing a detailed and nuanced insight into the community’s views on mental health issues.

### 2.2. Study Setting

The research was conducted in Matsafeni Village, situated 5 km from Mbombela city in Mpumalanga province, South Africa. The village, home to approximately 3723 residents across 826 households, is predominantly female (51%) and linguistically diverse, with 58% speaking IsiSwati, 29% speaking Xitsonga, 3% speaking Sesotho, and 3% speaking Afrikaans [23]. Despite having a private clinic providing basic healthcare, the village struggles with high unemployment rates and widespread adult illiteracy. The selection of this village was informed by the observed challenges faced by individuals with mental health disorders, as evidenced by their presence on the streets, appearing disoriented, lost, and disheveled.

### 2.3. Sampling and Participants

A convenience sampling method was used to select 15 community members or residents of Matsafeni village, with diverse demographics representing varied age groups, genders, occupations, and educational backgrounds. The study included residents of Matsafeni village. Sample size determination was based on data saturation, which is the point in research where new information or insights become limited, indicating that a comprehensive understanding of the topic has been achieved. The decision to stop data collection at the 15th participant was because no new information was coming forth except repetition. 

### 2.4. Data Collection Instrument and Procedure

Data were collected through unstructured interviews, with voice recorders capturing responses and field notes documenting non-verbal cues. In the study, participants were involved in one-on-one semi-structured interviews, where open-ended questions played a crucial role in exploring their understanding and perceptions of mental health disorders. The interviews covered various aspects, including causes, symptoms, available treatments, help-seeking behaviors, and community experiences related to mental health. Questions asked, which framed the interviews, included: “What do you know and think about mental health issues?”; “What makes people have mental health problems, and can they be treated?”; “Can you share your thoughts on how mental health is perceived within the Matsafeni community?”; and “How do cultural factors influence the way mental health is understood and discussed in Matsafeni?”.

The application of open-ended questions in this context allowed participants to express their thoughts and experiences freely, without being constrained by predefined answer options. The questions aimed to capture a broad range of responses, providing insights into the diversity of perspectives within the community. The open-ended nature of the inquiries encouraged participants to elaborate on their beliefs and experiences, fostering a more comprehensive understanding of their views on mental health. To enhance the richness of the data, the interviewers used probing and clarity-seeking questions. Additionally, a pilot study helped in identifying and addressing any potential cultural insensitivity in the questions. The feedback gathered from these pilot interviews was then utilized to refine the overall interview approach. Interviewers familiarized themselves with the study setting beforehand, gaining insights into the cultural norms and practices of the area. Collaboration with community leaders and influencers played a crucial role in establishing trust and legitimacy, seeking approval and guidance to ensure the research’s cultural appropriateness and respectfulness. Local language and communication styles were employed during data collection to foster a more comfortable and culturally sensitive interview environment. The researcher, maintaining a neutral, empathetic, and impartial stance, facilitated the interviews while taking comprehensive field notes. Bracketing and reflective remarks were employed to enhance the depth of insights.

### 2.5. Data Analysis and Measures of Rigor

All interviews were transcribed and analyzed using NVivo 24.23.0 software, ensuring systematicity and efficiency. The use of software facilitated the organization and retrieval of coded data, contributing to the rigor and accuracy of the analysis. A comprehensive coding framework was established, comprising an increased number of elements to code the identified categories and subcategories. The researcher and supervisors independently analyzed transcripts, and inter-coder reliability measures were employed to assess the consistency of coding decisions. Any discrepancies were discussed and resolved to enhance the overall reliability of the coding process. Regular joint meetings ensured consensus, fostering a robust and well-validated interpretation of the data. Thematic data analysis incorporated direct quotations for an authentic representation, adding depth to identified themes and sub-themes. 

Trustworthiness was ensured through credibility, transferability, confirmability, and dependability measures. Credibility was assessed through member checking, allowing participants to review and validate the findings to ensure accurate representation of their perspectives. To enhance transferability, the study provided detailed descriptions of methods, setting, and participants, enabling other researchers to assess the applicability of the findings to similar contexts. Confirmability was achieved by maintaining an audit trail, documenting decisions and steps during data collection and analysis, providing transparency for others to verify procedures. Dependability was strengthened through inter-coder reliability measures, with both the researcher and supervisors independently analyzing data. Thematic consistency was maintained through regular joint meetings, addressing discrepancies and fostering consensus on emerging themes.

### 2.6. Ethical Considerations

This study forms part of a broader research project on the perceptions and knowledge of Matsafeni Village community members regarding mental health disorders, approved by the Turfloop Research and Ethics Committee (TREC) at the University of Limpopo (clearance certificate number TREC/624/2022: PG). The Matsafeni Traditional Council (Sphezi Royal House) granted permission for the study. Participants signed written consent forms, acknowledging their voluntary participation and their right to withdraw at any stage without consequences. Privacy and confidentiality were meticulously upheld for participants and their personal information.

## 3. Results

Table 1 details the demographic backgrounds of the 15 study participants. The sample was balanced across age groups (six participants aged 18–35, five aged 36–50, and four aged 50–60). Marital statuses varied: four were married, four were single, two were cohabiting, one was in a relationship, one had never been married, and one was divorced. Employment status also varied: seven participants were self-employed in various capacities, five were employed, and three were unemployed.

Table 2 visually presents the identified themes and sub-themes derived from the interview data. The initial theme delves into the comprehension of mental health disorders, further categorized into three distinct sub-themes. The subsequent theme centers around the perceptions of mental illness, with three sub-themes providing a detailed exploration of participants’ perspectives on this topic.

### 3.1. Theme 1: Understanding of Mental Health Disorders

#### 3.1.1. Sub-Theme 1.1: Description of Mental Illness

Participants articulated mental health disorders as disruptions in thought patterns resulting in a detachment from reality. The described symptoms encompassed challenges in self-care, a sense of perceived irrationality, fluctuations in intelligence, memory lapses, intense feelings of sadness, and heightened excitement.

Participant No. 1: “Mental health disorders means that a person is failing to think normally”.
Participant No. 2: “Is when a person has a shortage of memory, he/she cannot think like any other person and requires care and patience”.
Participant No. 3: “Is when a person is bewitched and made to lose his/her mind”.

#### 3.1.2. Sub-Theme 1.2: Signs of Mental Illness

Participants recognized different signs and symptoms of mental health disorders, stressing the importance of spotting these signs. Some mentioned behaviors like people taking off their clothes in public, others always seeming hungry, or having strange conversations. Here are a few quotes from what participants shared:

Participant No. 3: “There are those that would just take off their clothes on the streets but for others, you cannot really tell that they are suffering from this illness if it is not extreme. People that are mentally ill are always hungry and eating continuously”.
Participant No. 4: “A person that is mental unfit can be seen rooming around the streets, untidy, staying on the streets, holding sharp things which they use to inflate pain on themselves”.
Participant No. 10: “The signs that I noticed from people that are affected by this illness involve poor communication (not making sense when talking, limited replies, giving irrelevant responses to questions asked or not responding at all), ignore other people and avoid communication with them, and they always seem like their minds are preoccupied”.

#### 3.1.3. Sub-Theme 1.3.: Behaviors of Mental Health Patients

The study discovered that people with mental illness go through various experiences within families where someone is diagnosed with mental health disorders. Two participants shared their personal experiences with family members diagnosed with mental health disorders, pointing out the difficulties, emotional stress, and ways of coping with supporting those affected. Here are some quotes from the interviews:

Participant No. 1: “Picking papers on the streets and eating from garbage bins”.
Participant No. 2: “Their behavior shows that they are mentally unwell. This can include them being very dirty, leaving their homes to stay on the streets, talking alone and sometimes arguing by themselves, and sometimes portraying signs of being disoriented. It can be easy to identify a mentally ill person in that they can be a danger to themselves and others and or insult others on the streets. This is a result of them not being able to differentiate between right and wrong.
Participant No. 13: “The sufferers of mental illnesses might throw themselves to the ground, hurting themselves and posing danger to other people”.

### 3.2. Theme 2: Perceptions of Mental Illness

#### 3.2.1. Sub-Theme 2.1.: Perception of Causes of Mental Health Conditions

Participants attributed mental health conditions to witchcraft, genetics, poverty, overthinking, substance abuse, and attempts at terminating pregnancies. This sub-theme captured diverse perspectives on the origins of mental health disorders.

Participants No. 2: “Mental illness results from when a person stole something from someone and they are bewitched, however, there are some people that are born with the illness”.
Participants No. 4: “In my view, there are two causes of mental health disorders, there are those that are born with the sickness and those that encounter changes due to parents using them to enrich themselves (black magic)”.
Participant No. 9: “For some kids is the shortage of cells required in their system, this can be resultant from a mother who tried to abort the baby during pregnancy, due to the intake of the foreign medications, the baby might be born with mental disabilities”.

#### 3.2.2. Sub-Theme 2.2.: Seeking Help for Management of Mental Disorders

Participants unanimously advocated for seeking help for mental health disorders, emphasizing the importance of treatment and support. Some participants suggested traditional healers and herbalists, while others recommended medical facilities and therapies.

Participant No. 4: “The mentally ill people can be assisted through admission into the mental institution, where government can fund and render the necessary treatment services for them to heal from their disorders. In these institutions, mentally ill people can be placed in the same place with other people that are mentally ill, taken through schooling processes; this will assist them in not feeling less of human beings. I think churches can also assist in curing the sicknesses as well as hospitals, depending on the type of illness the person suffers from”.
Participant No. 6: “People with mental illnesses must all possibly seek help they can get from traditional healers; this will assist in reducing the symptoms that comes with the disorder”.
Participant No. 14: “I believe people with mental illnesses should get help, from strong herbalist with vast indigenous traditional medicinal herbs and even clinics for them to stop hurting themselves and others”.
Participant No. 15: “I believe that mentally ill people must seek help through therapy, social workers and an established healthy environment which can also assist in the reduction of stress, as the illness will not just disappear without treatment”.

#### 3.2.3. Sub-Theme 2.3.: Perception on Treatment Options for Mental Health Conditions

Participants’ responses regarding treatment options varied, with some suggesting hospitals, medications, therapy, traditional healers, and herbalists. This sub-theme highlighted the diverse perceptions of the efficacy and appropriateness of different treatment modalities.

Participant No. 6: “The places where these people can be assisted are the hospitals, and from traditional healers. This may not completely cure the illness but it can reduce the symptoms”.
Participant No. 13: “I don’t know where people with mental health disorders can receive the help they require to heal through the western methods, but traditional healers have been helpful to mental health sufferers for a very long time”.
Participant No. 14: “They can get treatment from the hospitals through intake of medication or they can visit a strong traditional herbalist who has vast experience in the indigenous herbs”.

## 4. Discussion

Participants in this study suggested that symptoms of the existence of mental health problems, including challenges in self-care, perceived irrationality, fluctuations in intelligence, memory lapses, intense feelings of sadness, and heightened excitement, resonate with established diagnostic criteria and descriptions of various mental health conditions. These descriptions align with the World Health Organization’s (WHO) definition of mental disorders, characterized by abnormalities in thoughts, perceptions, emotions, behavior, and interpersonal relationships [1]. The Diagnostic and Statistical Manual of Mental Disorders (DSM-5) acknowledges the intricate interplay of cognitive and emotional dimensions in mental health disorders [24]. For instance, challenges in self-care and fluctuations in intelligence are consistent with the cognitive impairments often associated with conditions like schizophrenia or severe depressive disorders [25]. The mention of perceived irrationality aligns with the distorted thought patterns observed in disorders like psychosis. However, some participants expressed a more cultural perspective, viewing mental illness as being bewitched and emphasizing a supernatural element that may not align with mainstream psychiatric definitions. These differing conceptualizations highlight the complexity of interpreting mental health experiences. The acknowledgment of bewitchment as a potential cause reflects the rich cultural tapestry and diverse belief systems within the community. This finding underscores the importance of considering cultural contexts when examining mental health experiences. Cultural beliefs and traditions play a significant role in shaping individuals’ understanding of mental health, influencing their perceptions of causes, symptoms, and appropriate interventions [26]. 

The study reveals diverse community perspectives on behaviors linked to mental illness. While some participants focus on visible actions such as picking papers and eating from garbage bins, others highlight potentially dangerous behaviors, such as individuals throwing themselves to the ground. This diversity underscores the complexity of mental health experiences and the need for nuanced, context-specific approaches. The findings emphasize the importance of considering both visible and harmful behaviors in comprehensive mental health assessments, informing targeted interventions for daily functioning challenges and safety concerns [15]. Individuals grappling with conditions like schizophrenia or severe depressive disorders may exhibit behaviors indicative of cognitive impairments, impaired reality testing, and challenges in daily functioning, as highlighted by the participants [27]. 

The emphasis on the potential danger posed by mentally ill individuals to themselves and others corresponds with the literature on the increased risk of self-harm or harm to others among certain subsets of individuals with mental health disorders [28]. This underscores the importance of understanding and addressing the complexities of managing the safety concerns associated with severe mental illnesses. Furthermore, the study shed light on various behaviors associated with mental illness, such as antisocial actions, disorientation, and self-harm [29]. These behaviors not only serve as outward manifestations of internal struggles but also pose challenges for the community in comprehending, empathizing, and providing appropriate support. It is therefore recommended that targeted community education programs be implemented to enhance understanding of mental health disorders, dispel myths, and reduce stigma. These programs should focus on promoting empathy, fostering open conversations, and encouraging early intervention.

The literature also supports the idea that individuals’ perceptions of mental health disorders are influenced by cultural and societal factors [30]. The participants’ perspectives highlight the complex interplay of cultural beliefs and personal experiences in shaping their understanding of mental illnesses. There is a need for research studies exploring cultural variations in symptom expression, emphasizing the importance of considering cultural contexts in mental health assessments.

The identification of witchcraft, genetics, poverty, overthinking, substance abuse, and attempts at terminating pregnancies as contributing factors echoes studies emphasizing the socio-cultural determinants of mental health [31,32]. Cultural beliefs, such as attributing mental illness to witchcraft or black magic, are deeply rooted and play a significant role in shaping the understanding of mental health within communities [33]. 

The acknowledgment that some individuals might be born with mental illnesses aligns with the broader discourse on the complex interplay between genetic predisposition and environmental factors in mental health etiology [34]. This highlights the importance of understanding local beliefs and incorporating cultural competence in mental health interventions to bridge the gap between biomedical and culturally rooted perspectives [16]. Overall, the participants’ perspectives underscore the need for culturally informed and contextually sensitive mental health interventions that recognize and respect the diversity of beliefs surrounding the origins of mental health disorders. Integrating such cultural insights into mental health programs can contribute to reducing stigma, enhancing community engagement, and improving the effectiveness of interventions [35,36]. Establishing culturally informed support groups that reflect the diverse backgrounds of the participants can provide a safe space for sharing experiences, coping strategies, and mutual support. Participant insights can inform the structure and content of these groups, ensuring cultural relevance and sensitivity. Collaborating with mental health professionals can create accessible services tailored to the cultural context of the community. Participants’ insights can guide the development of outreach programs, counseling services, and mental health resources that specifically address the emotional repercussions identified in the study.

It is recommended that initiatives such as enhancing community awareness through educational programs, promoting early intervention strategies, and strengthening mental health services with a focus on collaboration between Western and traditional healing approaches be initiated. It is recommended to develop tailored educational workshops based on participants’ experiences and needs. These workshops should address specific aspects of domestic violence impact, coping mechanisms, and resources available within the community. The participants’ unanimous emphasis on seeking help for mental health disorders aligns with existing literature highlighting the importance of early intervention and appropriate support mechanisms for individuals experiencing mental health challenges [7]. 

While some participants advocate for admission to mental institutions and medical facilities, others propose seeking help from traditional healers and herbalists. These varied suggestions highlight the participants’ contrasting beliefs regarding the most effective avenues for addressing mental health concerns. These diverse perspectives underscore the cultural and individual variations in beliefs about what constitutes appropriate and effective treatment for mental health disorders within the community. The participants’ endorsement of conventional treatments reflects an acknowledgment of mainstream medical practices and psychiatric interventions. Hospitals, medications, and therapy align with established clinical approaches to mental health care that have a foundation in scientific research and evidence-based practices [37]. The acknowledgment of hospitals, medications, and therapy reflects the influence of Western biomedical models of mental health care, emphasizing the role of medical interventions and psychotherapeutic approaches [38]. 

On the other hand, the endorsement of traditional healers and herbalists as treatment options reflects a cultural and holistic approach to mental health. Moreover, the participants’ endorsement of traditional healers and herbalists for mental health support reflects the significance of cultural competence in mental health interventions. Traditional healing practices often incorporate cultural and spiritual elements, viewing mental health within a broader context that includes social, environmental, and spiritual dimensions [39]. The preference for these alternatives may stem from a belief in their effectiveness and a cultural trust in the healing abilities of traditional practitioners. The existence of these differing perspectives highlights the importance of recognizing and respecting diverse cultural beliefs and individual preferences when addressing mental health concerns. Integrating both conventional and traditional approaches in mental health interventions may enhance the accessibility and acceptability of services, promoting a more comprehensive and culturally sensitive approach to mental health care. 

Integrating traditional healing practices within mental health programs has been recognized as valuable in bridging the gap between biomedical and traditional perspectives [40]. Inter-sectoral collaboration is crucial to delivering psychosocial rehabilitation for individuals diagnosed with severe mental illnesses that result in substantial functional impairment [41]. Promote collaboration between the healthcare sector and other relevant sectors, such as social services and community organizations, to provide comprehensive psychosocial rehabilitation for individuals with severe mental illnesses. Design interventions that involve the entire family unit. Participants’ insights may reveal the importance of family dynamics in the aftermath of domestic violence. Family-centered approaches can include counseling, communication skills training, and resources for parents to support their children effectively. Work closely with schools to implement awareness programs informed by participants’ insights. These programs can educate teachers, administrators, and students about the signs of domestic violence, its impact on children, and how to create a supportive environment within the school setting. 

## 5. Study Limitations

The study’s primary limitation is the small sample size of 15 participants, potentially restricting the generalizability of the findings to the broader community. A larger and more diverse sample would improve the study’s representativeness. The study focused solely on Matsafeni Village in Mbombela municipality, Mpumalanga Province, limiting the applicability of the findings to other parts of the municipality or province. The absence of specific epidemiological data for the study setting underscores the importance of conducting a quantitative component to understand prevalence. Moreover, the study did not triangulate data from interviews and observations, leading to a lack of validation.

## 6. Future Prospects

Empowerment of the community by translating research findings into actionable initiatives. This could involve capacity-building programs, resource allocation for mental health services, and community-driven initiatives to foster a supportive environment. Targeted community-based interventions should be developed based on the study findings, encompassing mental health education programs, awareness campaigns, and tailored support networks that address the specific needs identified in the village. Collaborative initiatives are essential, involving local community leaders, organizations, and stakeholders to ensure cultural sensitivity and depth in future research. By extending the study’s geographic scope to include additional villages, towns, or provinces within Mbombela municipality or Mpumalanga province, a more comprehensive understanding of mental health perceptions across diverse communities can be achieved.

Longitudinal studies over an extended period are recommended to track changes in mental health perceptions, contributing to a dynamic and nuanced understanding of mental health awareness over time. Integrating a quantitative component in future research will complement qualitative insights and provide epidemiological data specific to the study setting, enhancing the understanding of mental health disorder prevalence. Furthermore, diversifying the study’s sample to include individuals from various demographics, occupations, and educational backgrounds will increase the external validity of the findings, ensuring a more representative portrayal of the broader community. Undertaking cross-cultural comparative studies with other regions or communities will identify variations and similarities in mental health perceptions, contributing to a broader understanding of regional or cultural influences on mental health awareness. Exploring the implications of the study for mental health policies at local and regional levels is crucial. Aligning study findings with existing policies and suggesting improvements can positively impact mental health support systems. Interdisciplinary collaboration is encouraged, involving experts from psychology, sociology, public health, and related fields to enrich perspectives and contribute to a holistic understanding of mental health awareness.

## 7. Conclusions

Community members provided in-depth insights into mental illnesses, elucidating their understanding of different disorders, ability to recognize signs and symptoms, personal interactions with diagnosed individuals, and observations of typical behaviors in those with mental health conditions. Participants also highlighted the communication difficulties, social withdrawal, and preoccupations faced by individuals with mental health disorders. The reported symptoms, behaviors, and potential dangers associated with mental health disorders correspond with existing research, emphasizing the complexity of managing safety concerns in individuals with severe mental illnesses. The findings highlight the importance of community education programs to enhance understanding, dispel myths, and reduce stigma surrounding mental health. Additionally, the study underscores the influence of cultural and societal factors on individuals’ perceptions of mental health, emphasizing the need for culturally informed interventions. The recommendations include targeted community education, early intervention strategies, strengthened mental health services, and collaboration between Western and traditional healing approaches. This comprehensive and contextually sensitive approach aims to foster a better understanding of mental health, promote community well-being, and improve support mechanisms for individuals with mental health challenges.

## Figures and Tables

**Table 1:** Demographic Background.

Participants Identifier	Age Group	Gender	Marital Status	Level of Education	Employment Status
Participant 1	50–60	Male	Married	Grade 11	Self-employed
Participant 2	18–35	Male	Single	Grade 10	Employed
Participant 3	35–50	Male	Married	Grade 2	Self-employed
Participant 4	35–50	Female	In a relationship	Grade 6	Self-employed
Participant 5	18–35	Female	Single	Grade 8	Self-employed
Participant 6	50–60	Female	Never married	Grade 5	Employed
Participant 7	50–60	Female	Divorced	Grade 4	Self-employed
Participant 8	36–50	Female	Married	Grade 8	Self-employed
Participant 9	18–35	Male	Married	Degree	Self-employed
Participant 10	18–35	Male	Single	Grade 12	Unemployed
Participant 11	50–60	Male	Married	Grade 5	Employed
Participant 12	18–35	Female	Married	Grade 8	Unemployed
Participant 13	36–50	Male	Cohabiting	Grade 4	Employed
Participant 14	36–50	Female	Cohabiting	Grade 4	Employed
Participant 15	18–35	Male	Single	Grade 12	Unemployed

**Table 2:** Themes and sub-themes that emerged from data.

Themes		Sub-Theme
Theme 1	Understanding of mental health disorders	1.1 Description of mental illness
		1.2 Signs and symptoms of mental illness
		1.3 Behaviors of mental health patients
Theme 2	Perceptions of mental illness	2.1 Perceptions on the causes of mental health disorders
		2.2 Seeking help for management of the condition
		2.3 Perception on treatment options for mental health conditions

## Data Availability

The insights presented in this study are derived from data gathered from participants in Matsafeni Village, located in Mbombela, Mpumalanga Province. Therefore, the data supporting the conclusions of this study is not publicly available.
